# Antileishmanial and Immunomodulatory Effect of Babassu-Loaded PLGA Microparticles: A Useful Drug Target to* Leishmania amazonensis* Infection

**DOI:** 10.1155/2018/3161045

**Published:** 2018-06-25

**Authors:** Mayara Cristina Pinto da Silva, Jefferson Mesquita Brito, Amalia dos Santos Ferreira, Andre Alvares Marques Vale, Ana Paula Azevedo dos Santos, Lucilene Amorim Silva, Paulo Vitor Soeiro Pereira, Flavia Raquel Fernandes Nascimento, Roberto Nicolete, Rosane Nassar Meireles Guerra

**Affiliations:** ^1^Laboratory of Immunophysiology, Department of Pathology, Center of Biological and Health Sciences, Federal University of Maranhão, 65080-805 Sao Luís, MA, Brazil; ^2^Oswaldo Cruz Foundation, Rondônia, 76812-245 Porto Velho, RO, Brazil; ^3^Oswaldo Cruz Foundation, Ceara, 61760-000 Eusébio, CE, Brazil

## Abstract

The immunological and the anti*-Leishmania amazonensis* activity of babassu-loaded poly(lactic-co-glycolic acid) [PLGA] microparticles was evaluated. The anti-*Leishmania* activity was evaluated against promastigotes or amastigotes forms, in Balb/c macrophages. The size of the microparticles ranged from 3 to 6.4 *μ*m, with a zeta potential of −25 mV and encapsulation efficiency of 48%. The anti-*Leishmania* activity of the PLGA microparticles loaded with the aqueous extract of babassu mesocarp (MMP) (IC_50_) was 10-fold higher than that free extract (Meso). MMP exhibited overall bioavailability and was very effective in eliminating intracellular parasites. MMP also reduced* ex vivo* parasite infectivity probably by the increased production of nitric oxide, hydrogen peroxide, and TNF-*α* indicating the activation of M1 macrophages. The overexpression of TNF-*α* did not impair cell viability, suggesting antiapoptotic effects of MMP. In conclusion, babassu-loaded microparticles could be useful for drug targeting in the treatment of leishmaniasis, due to the immunomodulatory effect on macrophage polarization and the increased efficacy as an anti-*Leishmania* product after the microencapsulation. These findings are of great relevance since the development of new drugs for the treatment of neglected diseases is desirable, mainly if we consider the high morbidity and mortality rates of leishmaniasis worldwide.

## 1. Introduction

Leishmaniasis is a protozoan infection that is transmitted by a sandfly bite to man and other vertebrates. According to the World Health Organization leishmaniasis is considered one of the most important neglected diseases, since it is endemic in 84 countries, with high prevalence in tropical areas of the world [1].

The currently available treatments for leishmaniasis have been considered unsatisfactory because of the high prevalence of parasite resistance and high toxicity [2–4]. For this reason, different groups have been investigating new approaches and novel treatments in an attempt to discover products and formulations with anti-*Leishmania* activity that are, at the same time, safe and effective, to be used instead of the conventional treatment or concomitantly as an adjuvant therapy [5–7].

Ethnobotanical data have shown the use of babassu mesocarp both as food and as medicine for the treatment of inflammation, gastric ulcers, rashes, and vulvovaginitis [8]. The aqueous extract of babassu mesocarp possesses several therapeutic effects already proven in preclinical trials such as healing [9, 10], antitumor [11–13], anti-inflammatory [14, 15], antimicrobial [16, 17], and immunomodulatory activity [14, 15, 17–20].

Guerra et al. [20] have demonstrated that the aqueous extract of babassu mesocarp, when combined with* Leishmania amazonensis* antigens, exerts immunomodulatory activity, increasing interferon-gamma (IFN-*γ*) production instead of IL-10, which usually occurs in Balb/c mice.

Delivery technologies are currently in use for sustained and enhanced delivery of phytoderived bioactive compounds in the pharmaceutical sector. According to previous studies [21–24], the PLGA formulations offer advantages for drug delivery and cell penetration as they are biocompatible, biodegradable, and stable in biological fluids and have been shown to protect the loaded compounds from degradation, resulting in a sustained release. Those systems are able to increase the stability and to reduce the amount of drug to be used, mainly for natural products. In addition, the PLGA systems enhance immunomodulatory responses since they can be targeted by antigen-presenting cells and are able to promote a good interaction with phagocytes [7, 21, 25].

Based on this and on other reports showing the therapeutic potential of babassu, the anti-*Leishmania* effect of the aqueous extract of babassu mesocarp encapsulated with poly[lactic-co-glycolic acid (PLGA)] aiming to develop new therapeutic strategies for the treatment of leishmaniasis was evaluated.

## 2. Material and Methods

### 2.1. Animals

Female Balb/c mice, 8-10 weeks old, weight 20-24 g, were obtained from the University of Campinas. During the study, the animals were maintained under controlled environmental conditions at the Animal House of the Federal University of Maranhão (UFMA), Sao Luis, Brazil.

Water and food were provided* ad libitum* throughout the experiment. All experimental procedures were conducted according to the guidelines of the Brazilian College of Animal Experimentation and were approved by the Ethics Committee of UFMA (Protocol No. 23115011476).

### 2.2. Preparation of Babassu Mesocarp Aqueous Extract (BAE)

Babassu mesocarp flour was obtained at our laboratory and stored in a plant extract collection. This product had been previously analyzed for authenticity, integrity, and purity by physicochemical assays and chromatographic techniques. The extract was prepared as previously described [20].

The babassu mesocarp aqueous extract showed a yield of 76%. The aqueous extract has the following composition: 0.51 mg/mL protein and 29.8 mg/mL total sugar, including monosaccharides, reducing sugars, aldoses and ketoses, and polysaccharides according to HPAEC analysis [13]. The extract contained 56% total polyphenols, including 55% phenolic acids and 1% flavonoids [17].

### 2.3. PLGA Microparticle Preparation

The microparticles were prepared by an oil-in-water or water-in-oil-in-water emulsion/solvent evaporation technique which allows the incorporation of the aqueous extract mesocarp [21, 22]. Two different batches of microparticles were obtained: unloaded microparticles (CMP; control) and microparticles loaded with the aqueous extract of babassu mesocarp (MMP; 300 *μ*L, 10 mg/mL).

The BAE was loaded into the microparticles as follows: the organic phase used for the production of the batches consisted of PLGA (100 mg; Purasorb^®^ PDLG 5002, Purac Biomaterials, Netherlands) with equal proportions of lactic and glycolic acid (50:50) dissolved in 10 mL dichloromethane. For the production of the MMP, the aqueous phase containing 300 *μ*L of the aqueous extract of babassu mesocarp (10 mg/mL) was poured into the organic phase and homogenized at 15,500 rpm for 2 min. The water-in-oil phase was poured into an aqueous phase containing polyvinyl alcohol as tensoactive agent (3%, 10 mL) (Mowiol^®^ 40-88, Sigma Aldrich, USA).

In order to conduct the uptake assay fluorescent microparticles were prepared by the addition of the dye 6-coumarin (0.15 *μ*g/mg, Sigma Aldrich, USA)] to the organic phase [23, 24].

### 2.4. Characterization of Microparticles

The microparticles were reconstituted in double-distilled water for the measurement of the mean particle size by dynamic light scattering technique (DLS) and the zeta potential (Zeta Sizer Nano ZS-Malvern Instruments, UK). Data represented as mean size of multiple runs (n= 5).

For elementary analysis, the microparticles were visualized and images were acquired with an Inspect S50-FEI scanning electron microscope equipped with an energy-dispersive X-ray spectrometer (EDX). Briefly, MMP and CMP suspension samples were placed on a mesh carbon grid for microscope observation.

Fourier transform infrared (FTIR) spectroscopy was used to analyze the possible interactions between the mesocarp with the PLGA copolymer used in the microparticles. Briefly, samples of CMP, MMP, and Meso (free babassu mesocarp aqueous extract) were mixed with potassium bromide (1:100-KBr) discs and the spectra were obtained with an FTIR spectrophotometer (Shimadzu, IR-Prestige) in the wavelength range of 4000-400 cm^−1^.

#### 2.4.1. Ultraviolet-Visible Spectrophotometry

The efficiency of entrapment of babassu mesocarp in the MMP microparticle was determined by ultraviolet-visible (UV-Vis) spectroscopy. The calibration curve of the babassu mesocarp aqueous extract was constructed using a solution of methanol: phosphate-buffered saline (PBS) (40:60%, v/v), pH 7.4, which was linear in the range between 78.125 and 10,000 *μ*g/mL. The UV-Vis spectrum of the mesocarp was obtained by scanning in the range of 199-900 nm using methanol: PBS (40:60%, v/v) as blank [6].

The following regression equation was derived from the calibration curve of the babassu mesocarp aqueous extract to evaluate the encapsulation efficiency:

(1) λ  280  nm:  y=0.0002x+0.0431,with  linearity  r2  of  0.9924.

#### 2.4.2. Determination of the Entrapment Efficiency of Babassu Mesocarp

The rate of entrapment of babassu mesocarp extract into the microparticles was determined by UV-Vis spectroscopy. Briefly, 20 mg of each microparticle batch (CMP and MMP) was weighed and dissolved in 500 *μ*L acetonitrile. The solvent solubilized the particles and the mesocarp extract was released from the polymer matrix. The tubes containing the solvent were kept open for evaporation and the resulting precipitate was suspended in methanol: PBS (40:60%, v/v). The preparation was filtered (0.22 pm) and read in a UV-Vis spectroscopy at 280 nm. The percent entrapment efficiency (%EE) of the BAE was calculated by the following as previously described [25]:

(2) %EE=Calculated  of  extract  weight  in  the  microparticlesTotal  extract  in  the  microparticle×100%

### 2.5. Uptake of Microparticles by Murine Peritoneal Macrophages

#### 2.5.1. Isolation of Murine Peritoneal Macrophages

Murine peritoneal macrophages from Balb/c mice, stimulated with 2 mL of a 3% thioglycolate solution (Merck No. 1081900500), were prepared aseptically. Peritoneal macrophages were suspended in supplemented RPMI medium (10% fetal bovine serum and 50 pg gentamicin) and the number of viable cells was determined by the Trypan blue (0.01%) exclusion method, in a Neubauer chamber, under a bright-field microscope at 400x magnification [26].

#### 2.5.2. Uptake Assay

Peritoneal macrophages [item 2.5.1] suspended in supplemented RPMI medium (5 x 10^5^ cells/100 *μ*L) were placed on round glass coverslips, in a 24-well plate, and incubated at 37°C for 2 h in a humid atmosphere with 5% CO_2_ for adhesion of macrophages. Nonadherent cells were removed and the adherent cells, consisting of 98% macrophages, are now incubated for 1 or 24 h with 100 pg/mL of each batch of microparticles (CMP, MMP, CMP6-coum, or MMP6-coum).

At the end, the supernatant of the cultures was aspirated and used to determine the production of cytokines, nitric oxide, hydrogen peroxide, and arginase activity. Noninternalized particles were removed by triple washing with PBS and the presence of microparticles in the cells was evaluated by counting 100 macrophages stained with May Grunewald-Giemsa [26] with light microscopy (magnification 1,000X).

The slides treated with the fluorescent microparticles (CMP6-coum or MMP6- coum) were fixed with Mowiol and examined under a fluorescence microscope at 1,000X.

#### 2.5.3. Hydrogen Peroxide Production

Peritoneal macrophages (200 *μ*L) in supplemented RPMI medium (10^6^ cells/mL) were incubated (2 h, 37°C, and 5% CO_2_) in microplates. At the end, the wells were washed twice with RPMI for removal of nonadherent cells. Adherent cells were incubated for additional 24 h with CMP, MMP, or Meso (100 pg/mL). The supernatant was discarded and 375 ng/mL dihydrorhodamine 123 diluted in PBS was added. The cells were then stimulated with phorbol myristate acetate (10 nM), 1 h at 37°C. The reaction was stopped and the supernatants were used to measure hydrogen peroxide production in a flow cytometer (Guava^®^ easyCyte 8HT Benchtop). A total of 5000 events were acquired for analysis using the FlowJo software.

#### 2.5.4. Cell Viability

Cell viability was evaluated by the MTT assay (3-(4,5-dimethylthiazol-2-yl)-2,5- diphenyltetrazolium bromide) (5 mg/mL; Sigma, USA). After 48 h of incubation, 10 *μ*L MTT was added to the wells and the plates were incubated for 3 h at 37°C in the presence of 0.5% CO_2_. The reaction was read at 540 nm.

### 2.6. Antileishmanial Activity

#### 2.6.1. Parasites

Promastigote forms (5X10^5^/mL) of* Leishmania amazonensis* (IFLA/BR/67/PH8) were obtained from 5-day-old stationary phase cultures. The protozoa solution was centrifuged (200 ×g for 15 min) and promastigotes were suspended in RPMI supplemented with 10% fetal bovine serum.

#### 2.6.2. Analysis of Molecular Interactions by Surface Plasmon Resonance (SPR)

The molecular interaction assay was carried on using the crude extract of* L. amazonensis* (150 *μ*g/mL, pH 5.5) as a ligand (acetate, pH 5.5) and the babassu mesocarp extract or pentamidine as analytes, at concentrations of 100, 50 and 25 *μ*g/mL.

Molecular interactions were analyzed by SPR in a Biacore T200 system (GE Healthcare Life Sciences) using a CM-5 sensor ship for immobilization of the ligand. The* Leishmania* extract was bound to the surface of the chip by amine coupling after activation of the dextran matrix with a mixture of 1-ethyl-3-(3-dimethyl-aminopropyl)-carbodiimide-HCl and N-hydroxysuccinimide. The amount of ligand immobilized on the surface of the sensor chip was predetermined based on the ratio between the molecular weight of the ligand and analyte as follows:

(3) Binding  capacity  RU:  MW  analyteMW  ligand×Immobilized  ligand  level

#### 2.6.3. Evaluation of Babassu Mesocarp Effect against Leishmania amazonensis Promastigotes

*Leishmania amazonensis* promastigotes (5X10^6^/mL) were incubated with different concentrations of the babassu mesocarp extract (500 - 62.5 *μ*g/mL), or CMP, or MMP microparticles (100 - 3.125 *μ*g/mL) or pentamidine (10 - 0.625 *μ*g/mL), used as positive control and compared to the negative control (culture medium). After 48 h of incubation at 37°C and 0.5% CO_2_, the number of parasites was determined in a Neubauer chamber, under a bright-field light microscopy (magnification 400X). The 50% inhibitory concentration (IC_50_) was determined by nonlinear regression.

#### 2.6.4. Evaluation of MMP Effect on Amastigote Infected Peritoneal Macrophages

Peritoneal macrophages (5X10^6^ cells/mL) were cultured during 2 h on sterile glass coverslips, in 24-well plates, at 37°C and 0.5% CO_2_. After incubation, nonadherent cells were removed and counted. The remaining cells were incubated, for 4 h, in the presence of promastigote forms of* L. amazonensis* at a proportion of 10 parasites/macrophage (33°C, 0.5% CO_2_). After the removal of free parasites, infected cells were treated, during 48 h, with the babassu microparticles (MMP, 100 *μ*g/mL), control microparticles (CMP), or pentamidine (5 *μ*g/mL).

The cell preparations were stained with Giemsa to determine the number of intracellular parasites. The number of parasites in 100 macrophages was quantified under a light microscopy at 1,000 x [26]. The infection index in the different groups was calculated using the formula previously described [27]:

(4) Infection  index=%  Infected  macrophages  ×Number  of  amastigotes  per  macrophages=Total  number  of  macrophages.

### 2.7. Nitric Oxide Production

The accumulation of nitrite, a stable end-product of nitric oxide, in supernatants was determined with standard Griess reagent [28].

### 2.8. Cytokine Levels in Macrophage Cultures

The levels of TNF-*α*, IL-6, and IL-10 were determined in the supernatants from infected macrophages cultures by ELISA, according to manufacturer instructions (eBiosciences, USA).

### 2.9. Statistical Analysis

In this study, all tests were repeated three times and the results are expressed as the mean ± standard deviation (SD). All data were compared by analysis of variance (one-way ANOVA), followed by the Tukey-Kramer test (for three or more groups) or Student t-test (two groups). Differences were considered significant when* p*<0.05. All data were analyzed with the GraphPad Prism 7.0 software.

## 3. Results

### 3.1. Physicochemical Characterization of Microparticles

Table 1 shows the size distribution, the zeta value, and the entrapment efficiency of the microparticles loaded with the BAE. This incorporation increased the diameter of the microparticles but did not interfere with their zeta potential.

The microparticles containing the formulations with 6-coumarin were always smaller than those without the fluorescent dye, irrespective of the presence of BAE.

#### 3.1.1. Microscopic Appearance of the Microparticles

**Figure 1:**
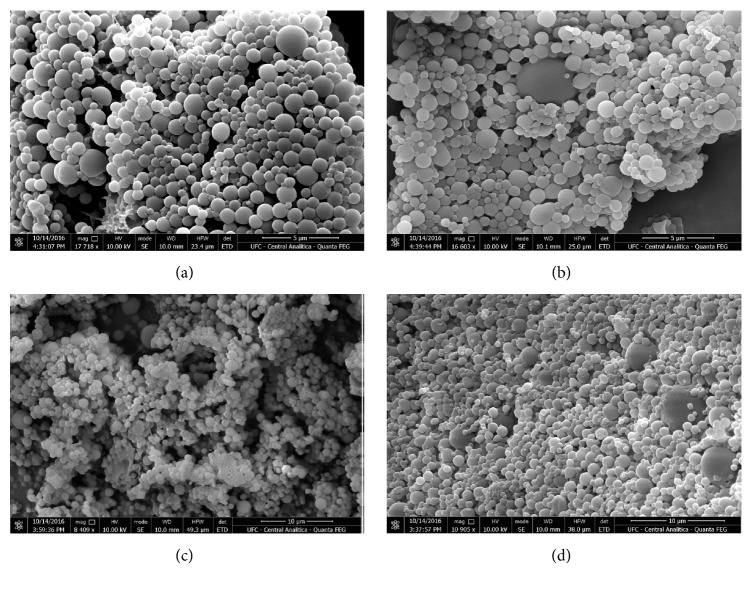
**Microphotography of the microparticles: (a-c) control; (b-d) microparticles loaded with babassu mesocarp.** For analysis, the microparticles were mounted on adhesive graphite tape attached to a metal support and sputter coated with gold. The observations were made at 10 kV under a scanning electron microscope equipped with an energy-dispersive X-ray spectrometer. Control microparticles containing 6-coumarin (c) and microparticles loaded with mesocarp + 6-coumarin (d) were also examined.

The scanning electron microscopy images clearly showed that the control microparticles (Figures 1(a)–1(c)) and those loaded with BAE (Figures 1(b)–1(d)) had a smooth surface and spherical shape and were almost homogenous, irrespective of the addition of 6- coumarin (Figures 1(c) and 1(d)).

#### 3.1.2. Babassu Interaction with Leishmania antigens and Evaluation of Entrapment of the Extract into Microparticles

**Figure 2:**
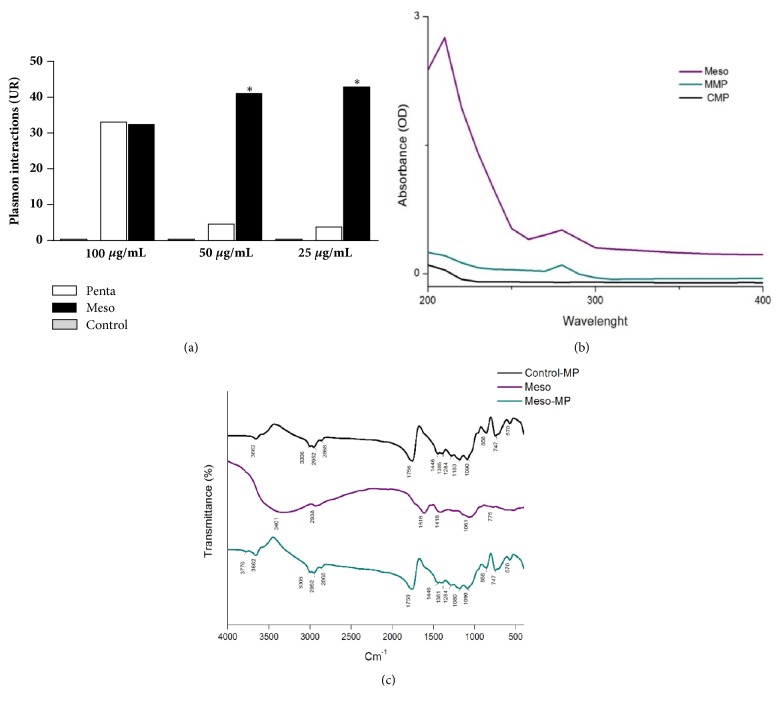
**Plasmon interaction of the aqueous extract of babassu mesocarp and of pentamidine with* Leishmania amazonensis* antigens (a), ultraviolet spectra (b), and FTIR spectra (c) of babassu-loaded microparticles (MMP)** compared to free babassu mesocarp extract (Meso) and unloaded (control) microparticles (CMP). For analysis of the molecular interaction by surface plasmon resonance, aqueous babassu mesocarp, or pentamidine (100, 50, and 25 *μ*g/mL) was bound to the ligand (*Leishmania amazonensis* crude extract, 150 *μ*g/mL, pH 5.5) immobilized on a CM-5 sensor chip. The ultraviolet spectra (b) show absorption of the samples at a wavelength of 200 to 400 nm. Individual samples were tested in triplicate and the data refer to one of the samples. (*∗*) p<0.05 compared to pentamidine. RU = resonance unit.

Analysis of the molecular interaction of the BAE with* Leishmania amazonensis* antigens showed a better interaction of the extract compared to pentamidine used as standard at the lower concentrations (50 and 25 *μ*g/mL) and also that the extract and the standard drug were similar at the higher concentration (100 *μ*g/mL) (Figure 2(a)).

The extract recovered after the dissolution of microparticles showed similar distribution ate 280-nm region and the same peak of absorbance as the free extract (Figure 2(b)). The FTIR spectra confirmed the efficiency of entrapment of BAE into the microparticles, as indicated by the observation of similar spectra and absorption bands for MMP and CMP, while different spectra were obtained for the free BAE (Meso), which exhibited several peaks in different regions and a broad and strong band in the 3,401 cm^−1^ region (Figure 2(c)).

#### 3.1.3. MMPs Are Less Toxic Than the Free Extract and Better Uptake

**Figure 3:**
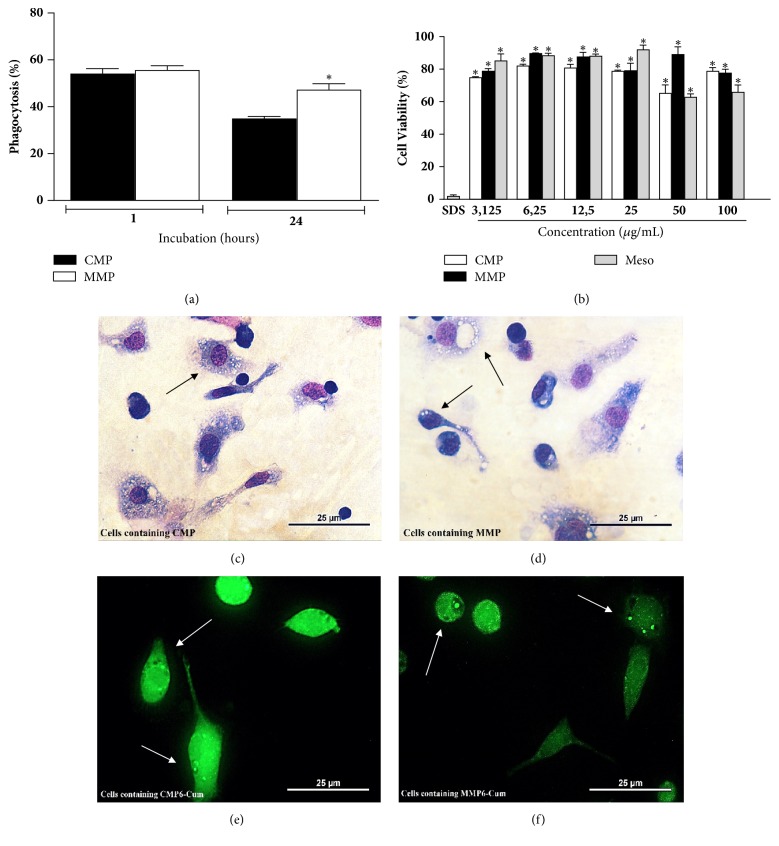
**Phagocytosis of babassu-loaded microparticles by peritoneal macrophages (a) and evaluation of microparticles cytotoxicity (b).** Uninfected peritoneal macrophages were treated for 1 h or 24 h with babassu-loaded (MMP) or unloaded (CMP) microparticles to determine the phagocytic index. In the cytotoxicity assays (b), sodium dodecyl sulphate-SDS (10%) was used as positive control and compared to macrophage cultures treated with babassu-loaded microparticles, unloaded microparticles, or aqueous extract of babassu mesocarp (Meso) at concentrations of 3 to 100 *μ*g/mL. Results are expressed as the mean ± standard deviation of individual samples tested in triplicate. (*∗*) p<0.05 compared to CMP (a) or SDS (b). The photomicrographs showing macrophages loaded with microparticles were obtained by light microscopy (c, d) or by fluorescence microscopy, using 6-coumarin incorporated into the two types of microparticles (CMP6-cum, MMP6-cum) (e-f).

Uptake of MMP was similar to that of CMP after 1 h of incubation. However, after 24 h of incubation, a 25% increase in the phagocytosis of MMP was observed compared to CMP (Figure 3(a)).

The MMP exhibited lower cytotoxicity than the free extract (Meso) at the higher concentrations. The results obtained for MMP were similar to those observed after treatment with CMP, with cytotoxicity ranging from 17 to 23%. As can be seen in the same figure, the higher doses of the extract were less cytotoxic when microencapsulated (Figure 3(b)).

It was possible to visualize the phagocytosis of the microparticles in the light (Figures 3(c) and 3(d)) and fluorescence microscopy photomicrographs (Figures 3(e) and 3(f)) due to the presence of several microparticles in the cytoplasm of murine peritoneal macrophages.

#### 3.1.4. MMP Exert Immunomodulatory Activity on Normal Peritoneal Macrophages

**Figure 4:**
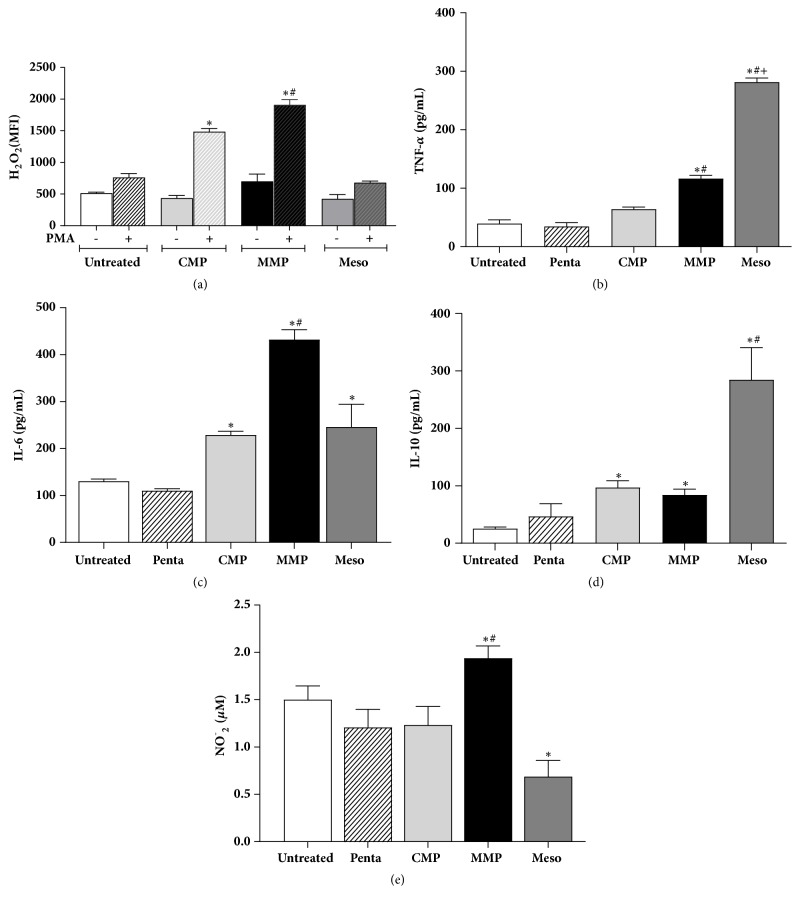
**Production of hydrogen peroxide (a), TNF-** **α** **(b), IL-6 (c), and IL-10 (d) by peritoneal macrophages incubated or not with microparticles.** The results obtained for uninfected and untreated macrophage cultures (untreated) were compared to those of cultures treated for 24 h with babassu-loaded (MMP) or unloaded (CMP) microparticles. Spontaneous production of hydrogen peroxide (H_2_O_2_) or production stimulated with PMA (a) was evaluated using dihydrorhodamine as fluorescent probe. The concentration of TNF-*α* (b), IL- 6 (c), and IL-10 (d) was evaluated by ELISA. Results are the mean ± standard deviation of individual samples tested in triplicate. (*∗*) p<0.05 compared to the untreated group; (#) p<0.05 compared to the CMP group.

Macrophages treated with MMP spontaneously produced more hydrogen peroxide than macrophages treated with CMP or than untreated macrophages. Stimulation with PMA potentiated the production of hydrogen peroxide in all groups, but higher production was always observed in the MMP group (Figure 4(a)).

Treatment of cultured macrophages with Meso or MMP increased the production of TNF-*α* (Figure 4(b)) and IL-10 production (Figure 4(d)) in comparison to the other two groups. The treatment with microparticles always increased IL-6 (Figure 4(c)), regardless of their content, when compared to untreated cells. Treatment with MMP increased the NO production in contrast with Meso that promotes a reduction in the production of this mediator.

### 3.2. Anti-*Leishmania* Effects of Microparticles

#### 3.2.1. MMP Was More Effective Than the Free Extract to Kill Promastigote Forms

The inhibitory concentration (IC_50_) of MMP (12 pg/mL) on promastigotes was, at least, 10 times lower than that found with the free extract (111.4 pg/mL) and, at least, 6 times lower than the CMP (71.8 pg/mL). Although it was 10 times higher than pentamidine (0.8 pg/mL), the MMP seems to be an effective anti-*Leishmania* product (Table 2).

#### 3.2.2. MMPS Were Effective against Amastigote Forms

**Figure 5:**
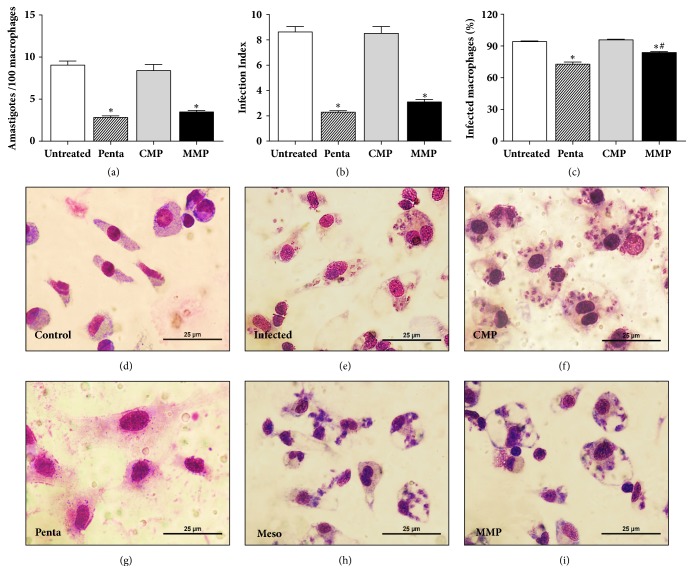
**Microparticles loaded with babassu mesocarp (MMP) reduced the amastigotes in infected macrophages.** The number of amastigotes in infected macrophages (a), infection index (b), and percentage of infected cells (c) in cultures treated with MMP were compared to those treated with the unloaded microparticles (CMP) or pentamidine (Penta). Results are the mean ± standard deviation of individual samples tested in quintuplicate. (*∗*) p<0.05 compared to the untreated and CMP groups. The light microscopy photomicrographs show uninfected macrophages (d), untreated infected macrophages (e), and infected macrophages treated with unloaded microparticles [CMP] (f), pentamidine [Penta, 5 *μ*g/mL] (g), aqueous extract of babassu mesocarp [Meso, 100 *μ*g/mL] (h), or babassu-loaded microparticles [MMP, 100 *μ*g/mL] (i).

Treatment of infected macrophages with MMP reduced the number of amastigotes with efficiency similar to pentamidine (Figure 5(a)). This anti-*Leishmania* effect was associated with a reduction on the infection index (Figure 5(b)). Treatment with MMP showed similar efficacy to pentamidine in reducing the number of infected macrophages (Figure 5(c)).

Figures 5(d)–5(i) show the images of the above results under light microscopy (400x) of normal macrophages (Figure 5(d)), untreated infected macrophages (Figure 5(e)), or infected macrophages treated with CMP (Figure 5(f)), pentamidine (Figure 5(g)), aqueous extract of babassu mesocarp (Figure 5(h)), or MMP (Figure 5(i)).

#### 3.2.3. The Immunomodulatory Effect MMP on the Ml Polarization of Infected Macrophages

**Figure 6:**
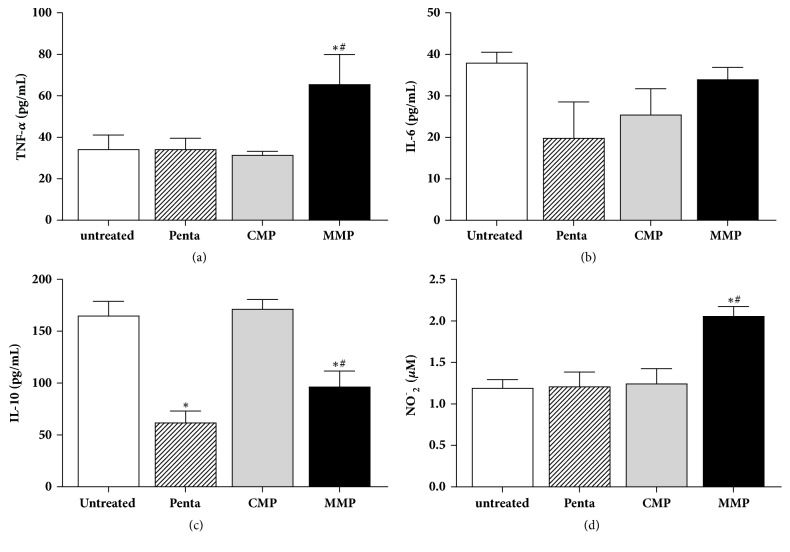
**Production of cytokines and release of nitrite in peritoneal macrophage cultures infected with** *Leishmania amazonensis*. Peritoneal macrophages infected with ***L. amazonensis*** were treated with 100 *μ*g/mL babassu-loaded microparticles (MMP), unloaded microparticles (CMP, negative control), or pentamidine (Penta, positive control). These groups were compared to untreated infected macrophages (untreated). After 48 h, the concentrations of TNF-*α* (a), IL-6 (b), and IL-10 (c) were evaluated in the supernatants by ELISA and nitrite production (d) was measured with the Griess reagent. (*∗*) p<0.05 compared to the untreated group; (#) p<0.05 compared to the CMP group.

MMP increased the production of TNF-*α* (Figure 6(a)) but had no effect on IL-6 levels (Figure 6(b)). On the other hand, MMP reduced the production of IL-10 (Figure 6(c)) similar to that seen in the group treated with pentamidine. In contrast, treatment with CMP showed no effect on cytokine production.

Treatment with MMP increased the production of nitric oxide compared to the other groups (Figure 6(d)). None of the treatments altered the activity of arginase (data not shown).

## 4. Discussion

In this study, we evaluated the anti-*Leishmania* activity of biodegradable microparticles loaded with the babassu mesocarp aqueous extract, the morphometric characteristics of those microparticles, and also their effect on macrophage activation. Microscopic particles can establish a long-term antigen release profile, enhancing the exposure of the antigens to antigen-presenting cells in the animals and consequently the induction of immune memory after challenge with the parasite [22].

The method employed allowed the production of microparticles with a desired diameter and zeta potential characteristics for a vaccine approach. In addition, the strong biomolecular interaction between the extract and* L. amazonensis* antigens suggests a possible therapeutic effect. At the lower concentrations, babassu mesocarp exhibited better interaction with* Leishmania* antigens than pentamidine, a standard drug, with anti-*Leishmania* activity, usually employed to treat* Leishmania* infections. Bioproducts that exhibit biomolecular interactions with microorganisms are important targets for bioprospection and should be used in the search for novel treatments since those biomolecular interactions may indicate an important effect on the microorganism [29, 30].

Various materials and structures have been employed as carriers for either passive or active targeting and among them poly(lactic-co-glycolic acid) (PLGA) microparticles. Those systems exhibit great potential for biomedicine as they are made of biopolymers approved by the FDA, with high biosafety, biocompatibility, and biodegradability [7, 21]. In addition, microparticles formulated with PLGA have shown wide applicability to drug delivery for different routes, and this system can sustain the delivery of lipophilic and hydrophilic drugs increasing their efficacy [21, 25, 31]. The efficacy of PLGA systems to drug delivery is associated with the charge reversal (cationization) in the acidic pH of endosomes that leads to localized destabilization of the membrane, fusion, and subsequent release of the particle and the drug into the cytoplasm, acting as a source of sustained release. To have a therapeutic effect, however, the chemical nature of the carried drug must be accounted for and the therapeutic effect of hydrosoluble drugs is more efficient [5].

In this context, the efficacy of microparticles produced with babassu as a new target to a drug design with anti-leishmania activity was evaluated. It was hypothesized that encapsulation of babassu mesocarp PLGA microparticles would increase their anti-*Leishmania *activity through increased cellular uptake, attenuated hydrolysis, and sustained release the extract in the cytoplasm. Thus, PLGA-loaded with babassu mesocarp aqueous extract (MMP) was designed, synthesized, and characterized and their anti-Leishmania effects were examined. We found that MMP formulation exhibited superior anti-leishmania effects compared to free extract (Meso).

The microparticles with babassu (BMP) or without babassu (CMP) have the diameter and the morphology biologically compatible to be ingested by the cells [22, 31–34], stability [34], a spherical shape, and a predominant homogeneous size, indicating adequate preparation of the formulations for biological purposes [32]. The FTIR analysis showed the efficient entrapment of the BAE, what is probably related to its chemical characteristics, since the surface of PLGA is hydrophobic and the extract as an aqueous solution is better carried by those microparticles [35].

The characterization of CMP and MMP by UV-Vis spectroscopy was compared to the aqueous extract of babassu mesocarp (Meso). Although two absorption peaks in the free extract in the ultraviolet region were found, the MMP spectra only exhibited one peak at the region of 280 nm, suggesting an association of this peak and the biological activity found to this microparticles. In addition, it is possible to use this peak as a marker of MMP microparticles, since this value is normally found for carbohydrates and proteins. In fact, 280 nm is a value to aldehydes, a functional group of almost all complex carbohydrates, as those found in babassu mesocarp.

The BAE contains in their composition: polyphenols, including phenolic acids and flavonoids [17] and carbohydrates, with the presence of monosaccharides, reducing sugars, aldoses and ketoses, and protein [13, 14]. These compounds may explain the efficacy of the babassu microparticles as an anti*-Leishmania* agent as demonstrated in the present study.

Previous studies have shown that aqueous babassu mesocarp extract induces both* in vitro *and* in vivo *nitric oxide (NO) and tumor necrosis factor *α* (TNF-*α*) production in peritoneal macrophages [15] and immunomodulatory activity after immunization with* Leishmania* antigens [20]. It is important to emphasize that the entrapment of the extract increased the anti-*Leishmania* activity and improved the safety of this microformulation, since a reduced toxicity of MMP was observed, mainly at the two highest doses.

The MMPs were more efficiently phagocytosed after 24 h of incubation confirming the efficient entrapment of the extract and suggesting a release of the extract over time, possibly due to degradation of the particles polymer, after contact with the acid pH of phagolysosomes.

Our hypothesis of macrophage activation and polarization is supported by the cytokine production, since the treatment with MMP increased the production of hydrogen peroxide, TNF-*α*, and IL-6 compared to CMP. In this study, we also speculated about the participation of signaling pathways, on the production of proinflammatory mediators produced during the macrophages' incubation (24 h) with the microscopic preparations. The incubation of macrophages with encapsulated BAE (MMP) resulting in high levels of TNF-*α* and IL-6, in the cell supernatants. It is known that activation of STAT3 signaling pathway results in an increased production of inflammatory mediators, including IL-6. This cytokine affects the lymphocyte proliferation and maturation and inflammation regulation [36, 37], corroborating the suggestion that microstructured preparations, especially those containing babassu, were able to stimulate macrophages, at the molecular level, for the production of an inflammatory response and the activation of M1 macrophages, instead of the M2 profile.

The protection or progression of the* Leishmania amazonensis* infection in mice models is related to the Th1 and Th2 activation, respectively, which can be characterized by the cytokine profile in each pathway [38], as well as the nitric oxide production in macrophages [39]. Infection control as a function of a Th1 response occurs through the elimination of parasites in macrophages by microbicidal mechanisms mediated by reactive oxygen species, such as nitric oxide (NO); in addition, TNF-*α* acts synergistically with IFN-*γ* to increase NO production by macrophages [18, 19].

Taken together, these results indicate that macrophage activation was mediated by the presence of the BAE and not by the microparticles. These results are in accordance with the observations of Nascimento et al. [15] who found an immunomodulatory effect of BAE on murine peritoneal macrophages, with an increased production of hydrogen peroxide and inflammatory cytokines. Cytokines such as TNF-*α* and IL-6 can modulate the immune response of macrophages, inducing an increase in the production of reactive oxygen species [40], as observed in our assays.

Macrophages that produce hydrogen peroxide and TNF-*α* and IL-6 have been characterized as M1 cells. The concept of M1 and M2 macrophages is based on the polarization of helper T lymphocytes (Th1/Th2). M1 macrophages are frequently activated in the presence of microbial products or proinflammatory cytokines [41]. These macrophages are characterized by an increased phagocytic activity and elevated production of proinflammatory cytokines such as IL-6 and TNF-*α*, as observed for macrophages treated with MMP, in addition to an increased production of nitric oxide.

It is important to note that both types of microparticles (CMP and MMP) increased the production of cytokines by macrophages activity that is probably related to the activation of NFk-B, even in the absence of any encapsulated compound [24].

Regulatory cytokines such as IL-10 and TGF-*β* inhibit macrophage activation, an event that is often associated with the aggravation of* Leishmania* infection. IL-10 protects the organism during chronic infections, reversing the intense inflammatory reaction [42, 43], impaired the microbicidal function and antigen presentation by macrophages and dendritic cells, and also inhibits the activation of Th1 cells [44] and M1 macrophages [45, 46]. Infected mice in the process of healing were treated* in vivo* with TGF-*β* and latent parasites were reactivated. Reactivation of the disease was accompanied by an increase in IL-10 production [47].

The entrapment of BAE potentiated by almost 10 times its* anti-Leishmania* activity against promastigote forms of* L. amazonensis*. Reports regarding the effects of the extract in solution on promastigotes are not new [48]. However, our study is the first to report the anti- Leishmania activity of microparticles loaded with babassu mesocarp.

The MMP was more effective against* Leishmania* than the BAE in solution when applied to cultures of previously infected macrophages that contained amastigotes in their cytoplasm. In general, infected macrophages are suppressed due to the increase in IL-10 and to the escape mechanisms of* L. amazonensis*, which result in a reduction of inflammatory cytokines and low production of reactive oxygen species [49]. Our results show that MMP exerted microbicidal activity against* L. amazonensis* and still preserved the immunological effect of babassu mesocarp, since phagocytosis of these microparticles reversed the suppression caused by infection. These findings suggest an immunomodulatory activity of these microparticles on the activation and polarization of M1 macrophages, in which an increased production of TNF-*α* and nitric oxide and, on the other hand, a reduced IL-10 production due to the slow release of the extract inside the cell was also observed. These results corroborate those of other studies regarding the immunomodulatory effect of BAE on the activation of macrophages [15], on the response of mice to* Leishmania* antigens [20], and on sepsis [17]. However, this is the first study showing the effect of babassu-loaded microparticles on the polarization of M2 to M1 macrophages during infection with* L. amazonensis*.

Unloaded microparticles exerted a moderate effect on* L. amazonensis* promastigote forms but no activity against amastigotes inside macrophages, indicating that the degradation of microparticles can interfere with the viability and/or proliferation of promastigotes by mechanisms that still need to be elucidated.

PLGA microparticles themselves cause cell instability and cytoplasmic changes due to their size [24], a characteristic that could explain their effect on promastigote forms. However, the presence of babassu mesocarp in the formulation potentiates cell activation, with a consequent increase in the production of chemical mediators and cytokines that contribute to the death and/or inhibition of proliferation of the protozoan in the cell cytoplasm.

High concentrations of inflammatory cytokines can exert a deleterious effect on cells, resulting in necrosis and/or apoptosis [50, 51]. It is important to emphasize that the entrapment of BAE reduced the effect on TNF-*α* and IL-10 production, a mechanism that may be associated with a better cellular efficiency and/or with a protection of the macrophages from death, what may be associated with the improvement on the anti-*Leishmania* activity of MMP in comparison to Meso. Thus, it is reasonable to propose that the moderate production of inflammatory cytokines induced by MMP not only ensures the reversal of immunosuppression caused by infection and the polarization of macrophages but also protects the cells from apoptosis without the risk of deleterious effects for the cell, even in the presence of increased production of TNF-*α*.

Considering the findings regarding the* anti-Leishmania* activity of MMP, we investigated whether secondary metabolites such as polyphenols are involved in the mechanism of this activity. Some compounds of the flavonol class inhibit the enzyme arginase, a therapeutic target of protozoa of the genus* Leishmania*, since this enzyme participates in pathways involved in the proliferation and differentiation of these microorganisms [52]. We evaluated the activity of arginase (data not shown) in cultures of infected macrophages treated with the two types of microparticles, but no inhibition was observed. Although indirectly, this result shows that polyphenols are not involved in the anti-*Leishmania* activity of MMP.

The results obtained revealed that the encapsulation of the aqueous extract of babassu mesocarp reduced the cytotoxicity of the extract for normal cells and potentiated its anti-* Leishmania* activity against both promastigote and amastigote forms. In addition, the encapsulated extract exerted immunomodulatory activity on murine macrophages by mediating the polarization of macrophages to the activated Ml phenotype.

The present results are of particular relevance if we consider that the large number of drugs used to treat leishmaniasis exhibits high toxicity and induces resistance of the parasite, resulting in therapeutic inefficacy and low treatment adherence, and that the polymer used for preparation of the microparticles (PLGA) has been extensively applied in models of drug release. PLGA is a biocompatible, biodegradable, and nontoxic polymer that does not require surgical intervention after depletion of the drug [53, 54].

In conclusion, the biodegradable microparticle formulation containing babassu mesocarp could serve as a prototype of new drugs for the treatment of leishmaniasis due to its direct action on promastigote and amastigote forms and its immunomodulatory effect on the macrophages polarization.

## Figures and Tables

**Table 1:** Diameter, zeta potential, efficiency of encapsulation, and polydispersion index of PLGA microparticles loaded with the aqueous extract of babassu mesocarp with or without 6-coumarin.

Microparticles^a^	Size^b^ (*μ*m)	Zeta potential^b^ (mV)	%EE_ _^c^	PDI
CMP	5 ± 0.8	−23 ± 0.8	NA^d^	0,401 ± 0,05
CMP6-coumarin	5 ± 0.6	−17 ± 0.6	NA	0,676 ± 0,11
MMP	6 ± 0.9	−26 ± 0.3	45 ± 5	0,703 ± 0,20
MMP6-coumarin	3 ± 0.2	−34 ± 1.8	42 ± 3	0,884 ± 0,05

(a) CMP: unloaded microparticles. MMP: microparticles loaded with the aqueous extract of babassu mesocarp. CMP6-coumarin: microparticles loaded with the fluorescent dye 6- coumarin and MMP6-coumarin: microparticles loaded with the aqueous extract of babassu mesocarp and the fluorescent dye 6-coumarin.

(b) Values are the mean ± standard deviation of samples tested in quintuplicate.

(c) EE: entrapment efficiency.

(d) NA: not applicable

**Table 2:** Minimum inhibitory concentration of babassu-loaded microparticles against promastigote forms of *Leishmania amazonensis* compared to unloaded microparticles and extract in solution.

Treatment_ _^a^	IC_50_ (pg/mL)_ _^b^
MMP	12^*∗*^
Meso	111.4
CMP	71.8
Pentamidine	0.8

(a) MMP: microparticles loaded with babassu mesocarp extract. CMP: unloaded microparticles; Meso: aqueous extract of babassu mesocarp in solution.

(b) IC_50_: 50% inhibitory concentration of individual samples tested in triplicate. (*∗*) p<0.05 compared to the other groups.

## Data Availability

The data used to support the findings of this study are available from the corresponding author upon request.
